# Crack-Free Precision during Desiccation: Optimizing Aerosol Jet Printing for High-Performance Conductive Microstructure Manufacturing

**DOI:** 10.1021/acs.nanolett.6c01881

**Published:** 2026-06-08

**Authors:** Geng Li, Yuxin Sun, Yanhong Tian, Jing Yu, Karol Viviana Mejia-Centeno, Malik Dilshad Khan, Shang Wang, Jordi Arbiol, Andreu Cabot, Qing Sun

**Affiliations:** † State Key Laboratory of Precision Welding & Joining of Materials and Structures, 47822 Harbin Institute of Technology, Harbin 150001, China; ‡ 235241 Catalonia Institute for Energy Research (IREC), Sant Adrià de Besòs, Barcelona 08930, Spain; § Catalan Institute of Nanoscience and Nanotechnology (ICN2), CSIC and BIST, Campus UAB, Bellaterra, Barcelona 08193, Spain; ∥ Faculty of Chemistry, University of Barcelona, Barcelona 08028, Spain; ⊥ ICREA Pg Lluis Companys, Barcelona 08010, Spain

**Keywords:** Aerosol jet printing, Desiccation cracking, Suction stress, Conductive microstructure, Additive manufacturing

## Abstract

Aerosol jet printing (AJP) is a high-resolution additive manufacturing technique that enables the deposition of conductive materials onto diverse substrates. However, a significant challenge in AJP is the occurrence of cracking in printed lines, which undermines both electrical conductivity and structural integrity. Herein, we examine the desiccation-induced cracking mechanism in AJP and introduce optimization strategies to mitigate cracking and enhance the conductivity of printed silver lines. Through a detailed analysis of crack morphology and distribution, key factors such as the deposition thickness and overspray regions are identified. The proposed optimizations, comprising regulation of the deposition thickness, enhancement of resolution, and minimization of overspray, yielded crack-free, high-quality silver lines with superior conductivity. These findings offer crucial insights for the advancement of reliable conductive structures in conformal electronics and microelectronics applications.

## Graphical abstract

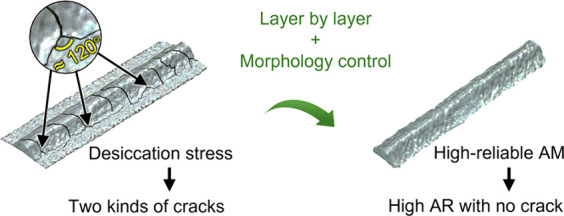

Aerosol jet printing (AJP) is an advanced noncontact, precision additive manufacturing (AM) technique that utilizes aerosolized ink for focused deposition,^1,2^ widely employed in the fabrication of micro/nanodevices and functional materials.^3,4^ AJP demonstrates remarkable advantages in material versatility, deposition resolution, and process adaptability, with the capability to achieve a minimum line width below 10 μm.^5^ It is applicable to flexible substrates, irregular surfaces, and intricate 3D geometries.^6,7^ AJP has been extensively utilized for high-precision patterning, enabling the deposition of multimaterial, multilayer structures, thus transcending traditional 2D fabrication.^8^ By meticulously controlling the nozzle-to-substrate distance, ink deposition rate, and curing parameters, AJP facilitates the precise layer-by-layer construction of self-supporting columns,^9^ microbridges,^10^ spatial interconnections,^11^ and high aspect-ratio 3D microcomponents.^12^ In comparison to conventional microfabrication or photolithography, AJP offers a streamlined, versatile, and scalable approach to the fabrication of complex 3D microstructures.

Whether for planar or 3D microstructures, the fundamental and smallest building unit remains the individual printed line.^13^ The geometric precision,^14^ morphological integrity,^15^ density,^16^ and electrical continuity of these lines directly govern the performance of the final deposited structure.^17,18^ Quality control of printed lines is a pivotal scientific and engineering challenge for realizing high-reliability AJP applications. Current research efforts to enhance line quality encompass several key areas: (i) aerosol stream dynamics analysis to suppress overspray effects, thereby improving line-width control and edge definition;^19^ (ii) the development of deposition morphology prediction and efficiency characterization methods to guide the regulation of 3D microstructures;^20^ (iii) the formulation of path planning strategies to accommodate intricate patterns and large-area deposition;^21^ (iv) the optimization of printing workflows and process windows for specific resolution or high-aspect-ratio structures.^22,23^ These advancements have significantly advanced AJP’s capabilities in resolution, structural complexity, and process stability.

However, we observed a recurrent cracking phenomenon with distinct characteristics in certain AJP-printed conductive silver lines during the experimental process. These cracks typically manifest at the scale of individual lines, displaying pronounced directional and spatial distribution patterns. Such cracking compromises the continuity of the conductive pathways, severely diminishing electrical performance.^24^ Furthermore, in multilayer stacking or 3D structure construction, these cracks induce stress concentration and structural instability, substantially limiting the reliable fabrication of both planar and 3D microstructures.^25^ More critically, these cracks are not random defects but rather consistently appear within specific processing windows, indicating the presence of an underlying, yet inadequately understood, formation mechanism. In contrast to issues such as overspray effects and line-width control, systematic investigations into the cracking behavior of AJP lines remain relatively scarce. Existing studies often regard cracks as incidental defects, lacking a thorough mechanistic analysis. This gap in understanding has, to some extent, hindered the further advancement of AJP in high-conductivity, high-reliability, and complex 3D structure fabrication.

Here we address the ubiquitous cracking phenomenon in AJP silver lines, employing a comprehensive analysis of the morphological characteristics of various crack types to deeply investigate their underlying formation mechanisms. Building on this mechanistic insight, we propose refined optimization strategies aimed at achieving the deposition of high-quality, high-conductivity silver lines. This study advances the fundamental understanding of line formation in AJP, introducing novel design and control paradigms for the reliable fabrication of both planar and 3D microstructures.

The morphology and structure of the silver nanoparticle (AgNP) ink materials are shown in Figure S1a,b. Under unoptimized AJP conditions, the printed lines exhibit typical cracking (Figure 1a), with the coexistence of transverse and longitudinal cracks. The width of transverse cracks shows a clear correlation with crack spacing: when the spacing is 55.6 and 47.0 μm, the crack width is 2.6 μm, whereas increasing the spacing to 83.0 and 71.3 μm enlarges the crack width to 4.4 μm. In brittle systems with constrained shrinkage, cracks form to release elastic strain energy accumulated from hindered volumetric contraction. Larger crack spacing allows greater strain energy accumulation between cracks, leading to greater energy release during propagation and thus wider crack openings. This indicates that crack formation is an energy-driven process caused by internal shrinkage stress rather than a random defect.

**Figure 1:**
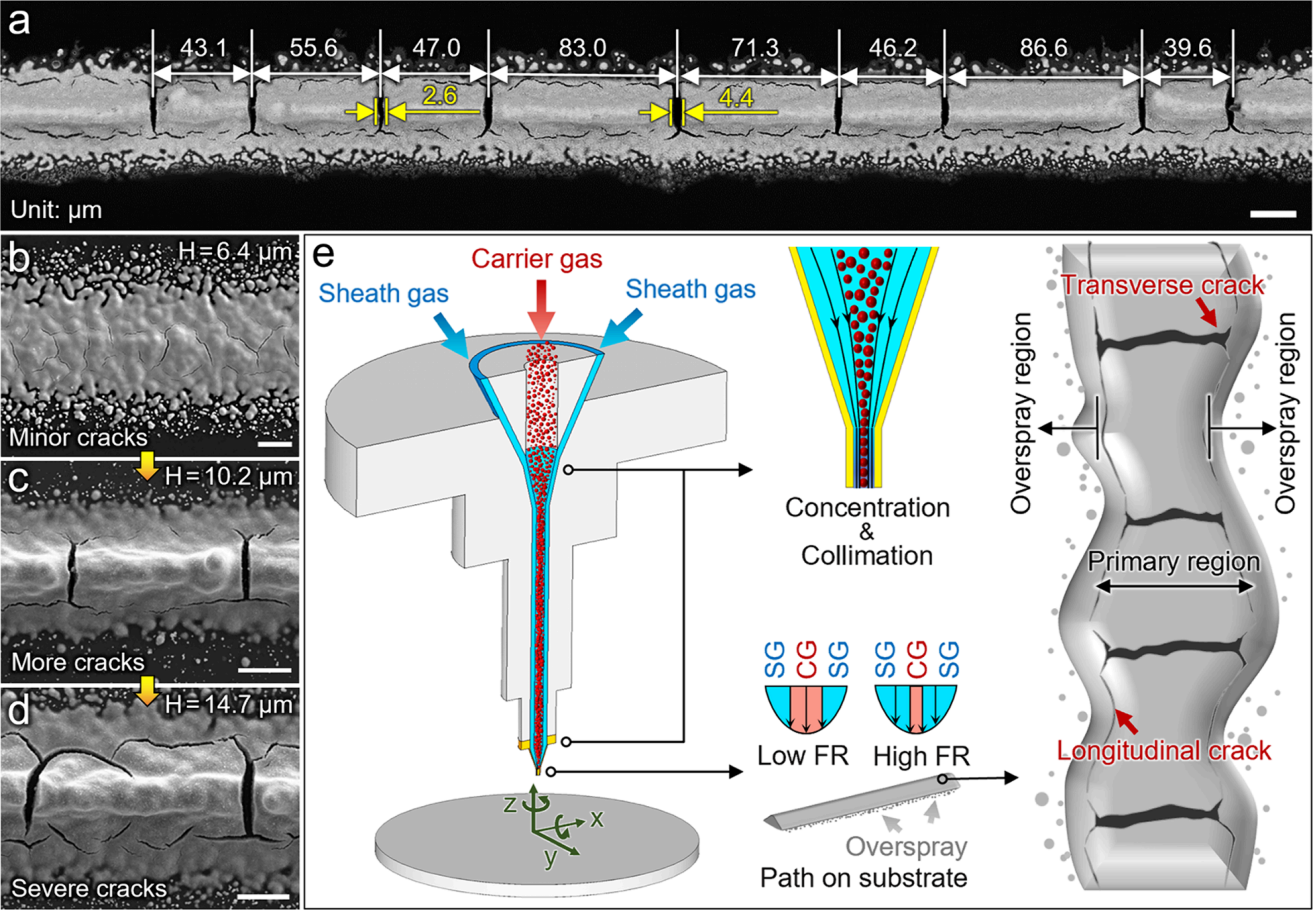
(a) SEM image of the cracked trace without optimization. (b–d) SEM images of the printed traces with thicknesses of 6.4 (b), 10.2 (c), and 14.7 μm (d). Scale bars: 20 μm. (e) Schematic diagram of AJP and the two types of cracks.

At the intersections of transverse and longitudinal cracks, a triaxial crack configuration with an angle of approximately 120° is observed. This morphology can be explained by both energy minimization and local stress equilibrium. In an isotropic plane stress state, multicrack systems tend to adopt an equiangular distribution to minimize total surface energy. For a three-branch crack junction, an equiangular configuration is energetically favorable when the fracture energy of each branch is similar. Under this condition, the three branches separate by approximately 120°, which minimizes the total energy of the junction. This interpretation is consistent with the energy-based analysis of shrinkage-induced star-shaped cracks reported by Gauthier et al.^26^ In addition to the energy-minimization argument, the 120° junction can also be understood from local configurational force balance. At a crack junction, each crack branch can be associated with a configurational force related to its fracture energy and propagation direction. For a mechanically stable three-branch junction, the vector sum of these configurational forces should vanish. This vector balance is satisfied when the three crack branches are separated by equal angles of 120°.

To evaluate the effect of deposition thickness on desiccation-induced cracking, silver lines with different heights were deposited in a single pass (Figure 1b–d). Transverse cracks first appeared at a height of 6.4 μm. As the height increased from 10.2 to 14.7 μm, both crack density and depth increased markedly (see quantitative results in Figures S2 and S3), exhibiting typical desiccation cracking behavior. This indicates that greater deposition thickness amplifies the evaporation rate gradient along the thickness direction, thereby increasing internal shrinkage stress and promoting crack propagation.

The AJP process and the associated cracking behavior are illustrated in Figure 1e. Micron-sized aerosol droplets carried by the carrier gas are focused by the sheath gas and expelled through a conical nozzle onto the substrate. Due to friction along the mist tube walls, the core aerosol stream attains higher velocity, while the Saffman lift force increases droplet density at the center,^27^ resulting in the highest deposition efficiency. Consequently, the main printed region exhibits a triangular or Gaussian-like profile, this kind of continuous central ridge with high thickness is defined as the primary region. Overspray, a common AJP defect, occurs when improper parameters or high-velocity droplets impact the substrate and splash, forming thin deposits on both sides of the primary region. These overspray regions do not enhance performance (e.g., conductivity) but instead reduce printing resolution, contaminate the substrate, and increase the risk of line bridging.

The crack morphology and distribution in the printed lines show clear patterns. Two crack types are observed: (i) longitudinal cracks, which have narrow gaps and run parallel to the printing trajectory, and (ii) transverse cracks, which are wider and perpendicular to the trajectory. Longitudinal cracks appear at the boundaries between the primary and overspray regions on both sides of the printing path, while transverse cracks are nearly evenly spaced and intersect the longitudinal cracks at their ends. Notably, these cracks are already present in the dried lines before sintering, indicating that they originate during aerosol deposition and subsequent drying rather than from the sintering process.

Aerosol droplets undergo substantial evaporation in the drying sheath gas,^28^ causing the solid content to increase from the initial ink concentration (typically 40–60 wt %) to a higher level before deposition. This promotes the aggregation of AgNPs into larger clusters, resulting in a deposit composed of solid AgNP aggregates and residual liquid. Owing to its high solid content and biphasic nature, the drying process resembles the desiccation of wet soil, where AgNP aggregates act as soil particles and the liquid phase corresponds to water.

Line cracking is closely related to evaporation or drying, and desiccation-induced cracking is largely governed by the solid–liquid phase ratio. However, in ink-based printing, the solid volume fraction, corresponding to the deposited solid after drying, is often unknown and much harder to determine than the mass fraction. In AM, deposition efficiency is usually evaluated on a volumetric basis because it directly determines the achievable 3D morphology and manufacturing process.

To further understand the desiccation-induced cracking of AJP-printed lines from the perspective of the deposition state, the actual solid deposition volume after ink drying was quantitatively characterized. The residue obtained from completely drying 0.2 mL of ink (Figure 2a) was analyzed using 3D X-ray computed tomography (XCT), revealing the overall morphology and internal structure from orthogonal directions. During drying in the cylindrical container, part of the liquid ink wetted and remained pinned to the inner wall of the container because of wetting, capillary adhesion, and contact-line pinning. As evaporation proceeded, the AgNP network gradually concentrated, shrank, and solidified. The shrinkage of this particle network generated fragmented and porous solid residues, Meanwhile, the thin liquid film initially attached to the container wall dried into ultrathin wall-adhered deposits. Figure 2b shows the overall scans, and Figure 2c presents cross-sectional slices along heights, where fragmented, porous, and ultrathin residues adhering to the tube wall are clearly resolved.

**Figure 2:**
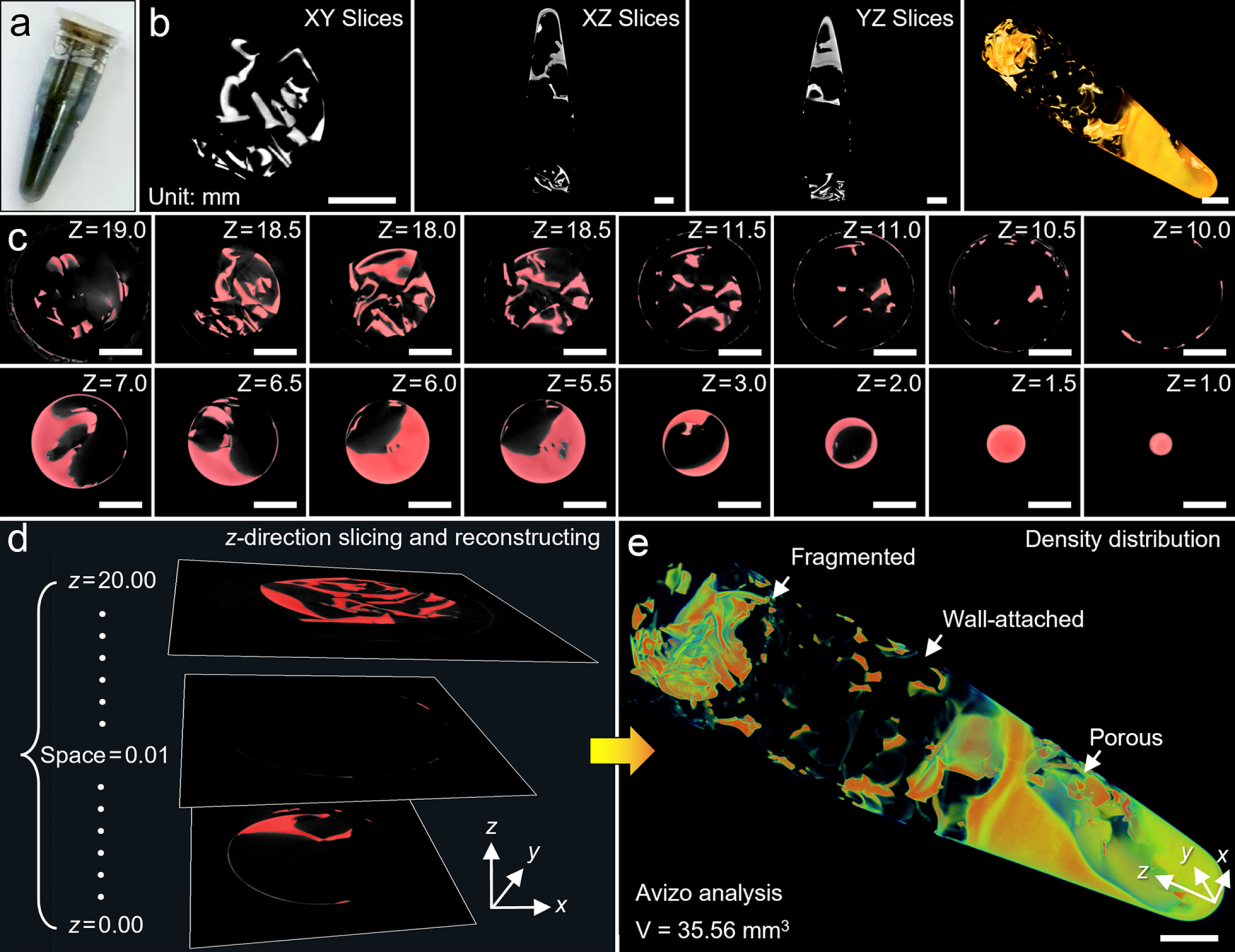
(a) 0.2 mL completely dried ink. (b–d) XCT analyses of AJP ink volumetric solid content. Scale bars: 2 mm. (b) Orthogonal slice views (*XY*, *XZ*, and *YZ*) and original 3D reconstruction. (c) Cross-sectional slices at different *Z* heights. (d) *Z*-direction slicing with a step size of 10 μm for 3D reconstruction. (e) Density distribution of the reconstructed structure.

Reconstruction of *Z*-direction slices with a 10 μm step (Figure 2d) produced the density distribution shown in Figure 2e. Avizo analysis determined a total solid volume of 35.56 mm^3^, corresponding to a volumetric solid content of 17.78%. This parameter directly reflects the deposited volume per unit ink volume and is therefore more suitable for evaluating deposition efficiency and 3D morphology design in AM. For AJP, where microstructures are built through droplet-by-droplet accumulation, the deposited volume directly determines line height, cross-sectional shape, and subsequent fabrication behavior.

Furthermore, under the focusing effect of the drying sheath gas, micron-scale aerosol droplets undergo significant and radially nonuniform evaporation: peripheral droplets may dry substantially, sometimes nearly completely, while central droplets shrink less. Although the exact volume change from atomization to deposition is difficult to quantify, a reduction in droplet radius to 70% of the initial value would raise the ink’s volumetric solid content from 17.78% to 51.84%. Under these conditions, liquid bridges and particle networks form, and the deposit no longer behaves as an ideal Newtonian droplet but as a concentrated biphasic system with finite yield stress and pronounced viscoplasticity, a high-solid-content, semiwet viscoplastic state that is deformable yet unable to freely level.

In this state, continued evaporation increases capillary suction, causing further contraction of the particle skeleton. When the surface solidifies earlier while the interior continues to shrink due to solvent loss, shrinkage mismatch and tensile stress accumulate, creating conditions for both transverse and longitudinal cracks. Thus, the 3D volumetric measurement not only quantifies the true volumetric deposition efficiency of the ink but also, from the perspective of solid-phase concentration in the deposited state, supports that AJP-printed lines constitute a desiccation-cracking system with a significant inherent cracking risk.

The drying process of the deposit can be divided into three stages, (i) constant rate, (ii) deceleration, and (iii) residual stages (Figure 3a), similar to the evaporation behavior of wet porous media such as soil or slurries. During the constant-rate stage, the surface remains covered by a liquid film, the liquid content is higher than the saturated content and evaporation is mainly governed by external conditions such as gas flow, temperature, and humidity. Because the atmospheric vapor pressure is lower than that at the deposit surface, solvent molecules continuously diffuse through the trace-atmosphere interface, sustaining a high and nearly constant evaporation rate. At this stage, the deposit remains fully wetted, with particles connected by liquid bridges and the structure not yet subjected to mechanical constraints.

**Figure 3:**
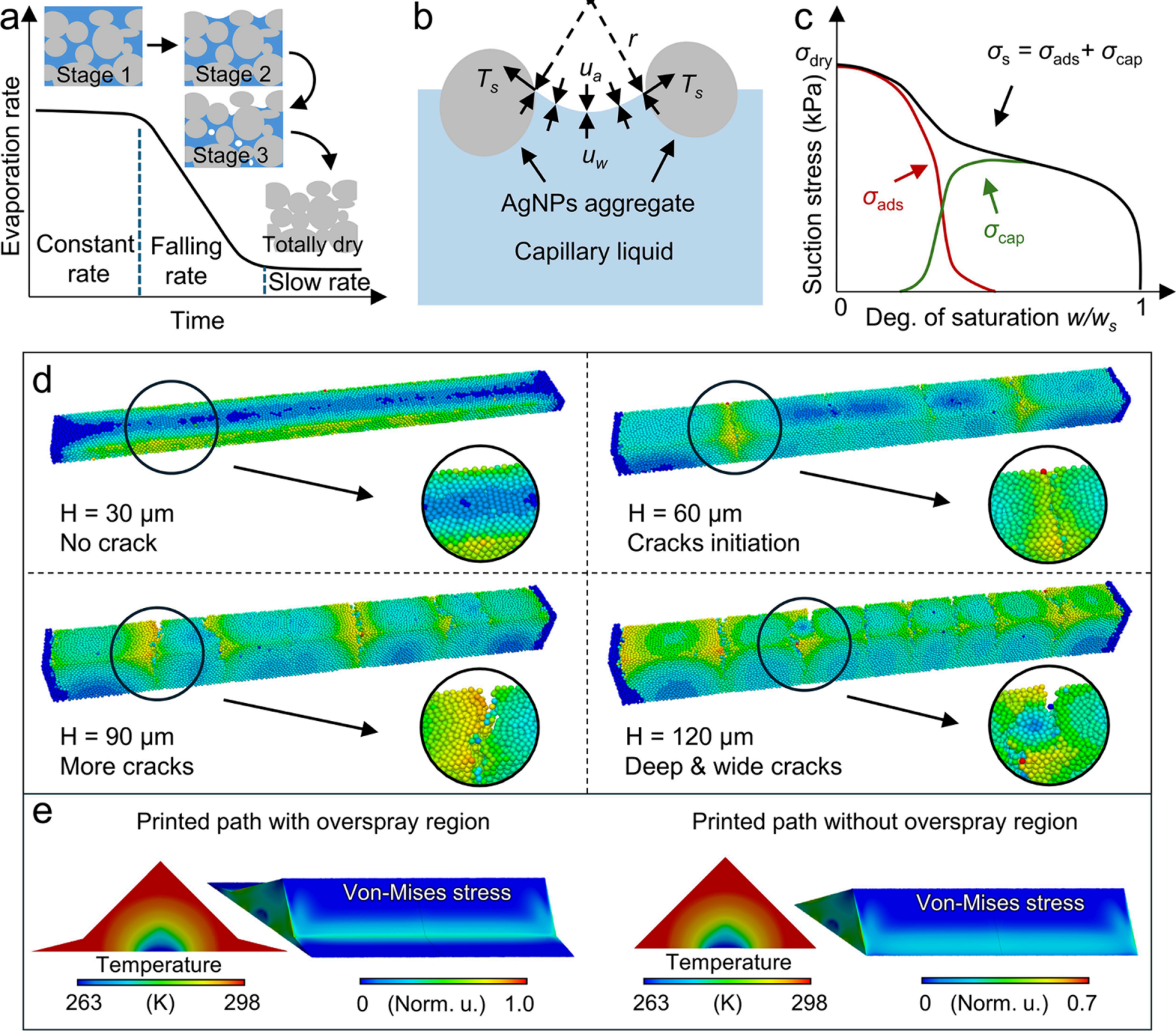
(a) Solvent evaporation process. (b) Capillary force. (c) Suction stress characteristic curve of printed traces via AJP. (d) Axial displacement of AgNP aggregates under different depositing thicknesses obtained by DEM simulation. (e) Temperature and stress distribution diagrams of different print features.

As evaporation continues, the deposit enters the deceleration stage, where the surface liquid film ruptures and the evaporation front recedes into the interior. Curved menisci form at the liquid–gas interfaces within the pores of AgNP aggregates (Figure 3b), generating capillary suction stress proportional to the liquid surface tension and inversely proportional to the meniscus curvature radius. This stress pulls AgNP aggregates closer, reducing pore size and causing macroscopic shrinkage. In the early part of residual stage, local drying inside the deposit produces partially emptied pores and locally dried cavities. These features can create additional menisci at remaining liquid bridges, so the capillary suction stress can further increase and reach a maximum. As drying continues, the remaining liquid bridges become thinner and finally disappear. Once the menisci vanish, capillary suction can no longer be sustained and gradually decreases to zero.

According to the unified effective stress theory proposed by Lu et al.,^29,30^ the total adhesive stress between particles arises from both capillary and adsorption forces (eqs S1–S6), with its evolution versus moisture content shown in Figure 3c. At high moisture levels, capillary suction dominates, whereas at lower moisture contents the adhesive stress approaches the macroscopic van der Waals attraction between AgNP aggregates. During this stage, the deposit gradually evolves into a mechanically continuous yet brittle solid framework, creating the conditions for crack initiation.

After identifying the source of internal adhesive stress during drying, the mechanism of crack initiation can be clarified. Cracking does not result from the absolute magnitude of adhesive stress but from differential evaporation within the deposit. Because the surface evaporates much faster than the interior, it dries first and forms a rigid “skin”. Meanwhile, the interior continues to evaporate and shrink, while the hardened surface layer restricts this contraction, generating tensile stress at the surface.

For an infinitely large isotropic deposit, once the suction stress exceeds the interparticle cohesive strength, cracks may initiate at multiple surface defects or local weak points and then propagate in different directions. The resulting crack network would tend to be randomly oriented rather than showing a single preferred orientation. However, the AJP trace has a high aspect ratio and is strongly constrained by the substrate, leading to the largest shrinkage along the length direction. Consequently, the primary tensile stress aligns with the printing trajectory. When this stress exceeds the tensile strength between AgNP aggregates, cracks propagate perpendicular to the principal stress, forming transverse cracks. Their position and spacing result from the balance between strain energy release and crack surface energy, producing a quasi-periodic pattern that minimizes total energy. Hence, the experimentally observed transverse cracks are approximately equidistant and represent periodic fractures in brittle deposits under constrained shrinkage.

Since transverse cracks originate from the evaporation rate gradient along the thickness direction, the discrete element method was used to analyze desiccation cracking under different deposition thicknesses. Increase of thickness leads to the gradual initiation of transverse cracks (Figure 3d), accompanied by a higher crack number, wider openings, and a quasi-periodic spatial distribution. This trend agrees well with the thickness-dependent observations in Figure 1b–d, confirming that greater thickness amplifies drying inhomogeneity and thus internal shrinkage stress. Accordingly, increasing the printing speed to reduce single-pass deposition thickness, combined with layer-by-layer deposition, is expected to suppress transverse crack formation while maintaining the overall thickness.

Unlike macroscopic systems, the spatial distribution of shrinkage stress in micron-scale printed structures is strongly influenced not only by thickness but also by printing morphology. During drying, this lateral heterogeneity results in nonuniform solvent removal and shrinkage. This difference in drying rate causes shrinkage mismatch between the primary region and the overspray region. In addition to the drying-rate mismatch, the shoulder between the primary region and the overspray region acts as a geometric discontinuity. which promotes stress concentration during constrained shrinkage. Finite-element analysis was further conducted to simulate drying shrinkage under low-temperature loading conditions and examine stress distributions for different morphologies (Figure S4). The results show that printed lines with pronounced overspray regions exhibit the highest stress levels (Figure 3e), with maximum stress concentrated at the interface between the primary deposition region and the overspray zone (Figure S5). This stress concentration indicates that longitudinal cracks originate from the geometric discontinuity at this boundary. Moreover, the simulations reveal that high aspect ratio patterns with less overspray exhibit lower overall stress, suggesting that minimizing overspray and improving printing resolution can reduce the risk of longitudinal cracking.

Thus, cracking in printed structures can be suppressed through two strategies: (i) using high-speed, layer-by-layer deposition of thinner unit layers to reduce drying inhomogeneity along the thickness direction and prevent transverse cracks; (ii) optimizing AJP parameters to minimize overspray and increase the aspect ratio of printed patterns, thereby avoiding stress concentration and longitudinal cracking. This mechanism-based strategy provides clear guidelines for fabricating crack-free, high-quality conductive structures via AJP.

Building on the understanding of desiccation cracking in AJP silver lines and its mitigation strategies, we experimentally validated the proposed mechanisms. For silver lines with a target height of approximately 15 μm (Figure 4a), unoptimized printing produced distinct transverse and longitudinal cracks. High-speed, multipass deposition reduced the single-layer thickness and effectively suppressed transverse cracks (Figure 4b), although longitudinal cracks persisted due to overspray regions. Further optimization, following our previous work,^31^ involved precise backpressure control to reduce overspray and increase the line aspect ratio (Figure 4c), yielding crack-free, high-quality lines. These results confirm that transverse cracks are mainly governed by the thickness-direction shrinkage gradient, whereas longitudinal cracks originate from lateral morphological discontinuities (Figure 4d).

**Figure 4:**
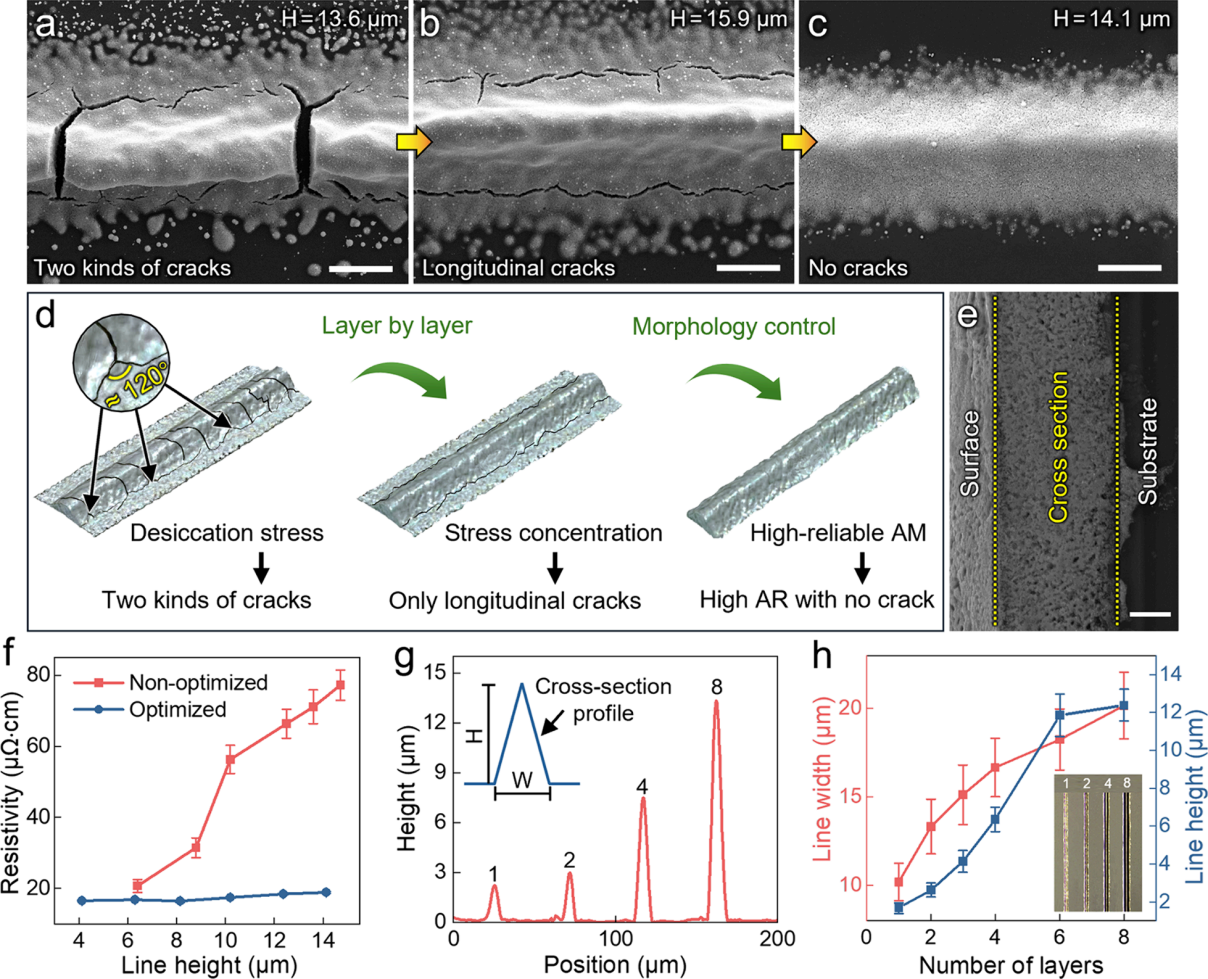
(a–c) Optimization process from two kinds of cracks (a) and longitudinal cracks (b) to no crack (c). Scale bars in parts a–c: 20 μm. (d) Schematic diagram of the cracking mechanism and optimizing process. (e) Cross-sectional view of printed line (scale bar: 5 μm). (f) Resistivities of printed traces. (g and h) Cross-sectional profile (g) and dimensions of high-resolution traces (h) with different printing layers.

The surface and cross-sectional views of the printed structures (Figures S6a,b and 4e) show crack-free and generally dense morphologies. To assess compatibility with different post-treatments, both thermal sintering and pulsed-light sintering (PLS; Figure S7a–c) were applied. After 90 min of thermal sintering, the microstructure evolved with temperature (Figure S8a). At 100 °C, the AgNP size remained unchanged and electrical transport occurred mainly through Ohmic contact between particles; undecomposed organic capping agents and numerous contact interfaces led to a resistivity about 1000 times that of bulk silver (Table S1). Increasing the temperature to 150–200 °C promoted particle growth due to the high surface energy of AgNPs, where enhanced atomic diffusion induced grain boundary migration and Ostwald ripening, eliminating smaller particles and enlarging larger ones. The combined effects of organic decomposition, neck formation, and reduced contact interfaces caused an exponential decrease in resistivity (Table S1). At 300 °C, the AgNPs fully sintered into a cohesive mass with much larger grains, producing a dense, smooth structure without observable pores.

The conductive structure after PLS treatment is shown in Figure S8b. At 400 V, AgNPs remain discrete and spherical, with large contact resistance, resulting in a resistivity exceeding 1 Ω·cm (Table S1). When the voltage exceeds 500 V, AgNPs begin to sinter and grow, and the conductive film becomes progressively denser and smoother. At 650 V, the particles fully coalesce into a smooth film, achieving a resistivity of 6.8 μΩ·cm, only 4.25 times that of bulk silver.

From a microscopic perspective, both thermal sintering and PLS, under suitable conditions, effectively bond AgNPs and form robust sintering interfaces (Figure S9a,b). This indicates that the electrical performance of printed patterns is mainly governed by crack suppression rather than the sintering method itself. Using thermal sintering as a baseline, we measured the resistivity of silver lines with different heights after sintering at 200 °C for 1 h (Figure 4f). For unoptimized lines, resistivity increased with thickness due to crack-induced disruption of conductive pathways (see dimensions of the test samples in Figure S10a,b). In contrast, optimized lines maintained consistently low resistivity across different thicknesses. At a thickness of 14 μm, the optimized lines maintained low resistivities of approximately 17 μΩ·cm over the investigated line-height range, showed only 24% of the resistivity of unoptimized samples, corresponding to about 10 times the resistivity of bulk Ag, highlighting the critical role of crack suppression in improving conductivity.

The mechanism-driven optimization strategy was further applied to ultrahigh-resolution printing for microelectronics packaging, interconnects, and flexible sensors. Using a 150 μm nozzle and layer-by-layer deposition, high-resolution line cross sections were achieved (Figure 4g). As shown in Figure 4h, the line width and height vary with the number of printed layers; with widths below 20 μm, aspect ratios above 0.65 were obtained. These results show that precise control of shrinkage behavior and printing morphology enables AJP to produce crack-free, highly conductive lines and supports the AM of arbitrary 3D conductive microstructures. Integrating heated substrates or in situ sintering/cross-linking could further extend this approach to more complex 3D conductive structures.

Crack-free, high-quality traces are essential for high-precision AM of conductive microstructures.^32–34^ Leveraging the noncontact and high-adhesion characteristics of AJP (Figure S11), large-area, customizable, stretchable, and flexible conductive patterns can be printed on various planar substrates, such as polyethylene terephthalate, glass, polydimethylsiloxane, and polyimide (Figure S12a–d), as well as on regular and irregular 3D surfaces (Figures S13a,b and S14). This capability enables precise fabrication of conductive patterns on both flat and curved substrates, supporting customized electronic components on complex 3D structures and flexible platforms. The understanding of desiccation cracking and the proposed optimization strategies address key quality and reliability challenges in AJP, offering practical guidance for high-quality patterning with high-solid-content inks or pastes.

Overall, we systematically investigated desiccation cracking in AJP and proposes effective suppression strategies. Deposition thickness and overspray regions are identified as key factors driving crack formation in silver lines. By optimizing printing parameters, reducing single-pass thickness via multilayer deposition, and minimizing overspray, both transverse and longitudinal cracks were effectively suppressed. Increasing the aspect ratio and improving printing resolution further enhanced the mechanical integrity and electrical performance of the lines. The study also clarifies the influence of printing and curing conditions on electrical properties, establishing a clear link between crack suppression and improved conductivity. Applying these strategies to ultrahigh-resolution printing enabled conductive lines with widths below 20 μm and aspect ratios above 0.65. These advances improve the reliability of printed conductive structures and provide new routes for fabricating complex 3D conductive microstructures for applications in microelectronics packaging, conformal electronics, and smart sensors.

## Supplementary Material



## Notes

Additional experimental details on materials, AJP procedures, characterization methods, conductivity measurements, PLS, and numerical simulations, together with supplementary figures and tables supporting morphology, cracking behavior, stress analysis, and the electrical performance of the printed structures (PDF)
