# Supplementary material for: Crack-Free Precision during Desiccation: Optimizing Aerosol Jet Printing for High-Performance Conductive Microstructure Manufacturing
Source: Nano Lett. 2026 Jun 8;26(23):7777–85. doi: 10.1021/acs.nanolett.6c01881 (PMC13281531; doi:10.1021/acs.nanolett.6c01881)
Supplement: Supplementary file 1 [file nl6c01881_si_001.pdf]

Supporting Information

Crack-Free Precision During Desiccation: Optimizing Aerosol Jet Printing for High-Performance Conductive Microstructure Manufacturing

Geng Li[a,b], *Yuxin Sun*[a], *Yanhong Tian*[a,*], *Jing Yu*[b,c], *Karol Viviana Mejia-Centeno*[b,d], *Malik Dilshad Khan*[b], *Shang Wang*[a,b], *Jordi Arbiol*[c,e], *Andreu Cabot*[b,e], *Qing Sun*[a,b,*]

[a] State Key Laboratory of Precision Welding & Joining of Materials and Structures, Harbin Institute of Technology, Harbin 150001, China

[b] Catalonia Institute for Energy Research (IREC), Sant Adrià de Besòs, Barcelona 08930, Spain

[c] Catalan Institute of Nanoscience and Nanotechnology (ICN2), CSIC and BIST, Campus UAB, Bellaterra, Barcelona 08193, Spain

[d] Faculty of Chemistry, University of Barcelona, Barcelona 08028, Spain

[e] ICREA Pg. Lluis Companys, Barcelona 08010, Spain

* Corresponding author: Y. Tian (tianyh@hit.edu.cn); Q. Sun (qsun@irec.cat)

Materials and Methods

Technological Process of AJP

A commercial silver nanoparticle ink (JSA221-AE) was obtained from Novacentrix (USA). The ink exhibits a viscosity in the range of 10-20 cp, making it well suited for aerosol generation using an ultrasonic atomization process. All printing experiments were performed using an aerosol printing system (PJ5X) from Neotech (Germany). The atomizer voltage was set at 40 V constantly. Purified compressed dry nitrogen stored at 25 °C was used for both sheath and carrier gases. All samples were printed with a nozzle of 250 μm inner diameter, a working distance of 2 mm and the glass substrates in room temperature.

Characterization

After printing, the samples were placed in 25 °C and 30% RH for 1 h to ensure complete drying. The microtopography of printed traces was characterized by scanning electron microscopy (SEM, Sigma 360, Zeiss, Germany). The cross-section profiles were obtained by laser microscope (VK-X150, Keyence, Japan). The conductivity was measured with a digital multimeter 34465A (Keysight, USA) using 4-wire mode. The samples consisted of a straight trace connected to electrode pads at both ends. The measurement was conducted across the entire printed trace, including the cracks. The resistivity data of printed trace was calculated by its resistance and cross-sectional area. The volume of the solid residues obtained after complete drying of the ink were characterized using a high-resolution X-ray micro-CT system (N80, NEOSCAN, Belgium). For the measurement, 0.2 mL of ink was placed in a cylindrical container and allowed to dry completely under ambient conditions. The resulting solid deposits were then subjected to micro-CT scanning. For quantitative analysis, the reconstructed datasets were imported into Avizo (Thermo Fisher Scientific). The solid phase was segmented from the background using grayscale thresholding, and the total solid volume was calculated using the built-in volume quantification module.

PFC Simulation

The DEM numerical simulations were performed using the 3D Particle Flow Code (PFC3D 6.0). Spherical particles were randomly generated to achieve a target porosity of 0.36. In the initial stage, particles interacted through a linear elastic contact model with an elastic modulus of 1.0 GPa and a

normal-to-shear stiffness ratio of 1.0. The friction coefficient was set to zero to mimic the lubricated, fully saturated state of the wet ink. The system was equilibrated until the unbalanced force ratio fell below 4×10^{-3}. Drying-induced shrinkage was simulated by switching to a linear parallel bond model to represent the formation of a solid particle network during solvent evaporation. The elastic modulus was reduced to 10 MPa. Shrinkage was implemented through a custom function: $S(z) = S_0 + k\frac{z}{h}$, where *S(z)* is radius shrinkage factor of particles at height *z*, S_0 is base shrinkage factor, *k* is shrinkage gradient and *h* is the height of the printed line. By gradually reducing particle radii with a thickness-dependent gradient, capturing non-uniform drying across the line thickness. Crack initiation and propagation were continuously monitored during incremental shrinkage steps, each followed by stress relaxation to mechanical equilibrium.

Finite Elements Simulation

Finite element simulations were conducted using ANSYS Workbench 14.0. The printed trace was reasonably simplified as a prismatic geometry with a cross-sectional shape comparable to the experimentally observed line morphology. To represent the surface stiffening caused by rapid solvent evaporation and early-stage crust formation, fixed mechanical constraints were applied to the outer surface of the printed line. Owing to the non-uniform evaporation rate, a moisture-content gradient develops predominantly along the direction normal to the free surface. This effect was equivalently modelled by imposing a temperature gradient across the line thickness.

Specifically, during drying, solvent evaporation is faster near the exposed surface than in the interior of the printed line. This non-uniform evaporation produces a through-thickness moisture-content gradient, which leads to differential shrinkage and the accumulation of stress. To reproduce this effect in a simplified but physically representative manner, low-temperature loads were applied at locations with equal distances from the outer surface to construct a controlled thermal gradient. Shrinkage induced by thermal contraction of the material under reduced temperature was used as an effective analogue for suction stresses generated during drying. The stress distribution with different morphologies were calculated to elucidate the influence of printing geometries.

The material properties of bulk silver, and a mesh convergence study was performed to ensure numerical accuracy. An indirect thermo-mechanical coupling approach combined with transient

analysis was adopted, in which a prescribed temperature field was used to mimic drying-induced shrinkage and to evaluate the stress distributions in printed traces with different morphologies. The initial temperature of the model was set to 298 K. A low-temperature convective boundary condition corresponding to 198 K was applied to the bule bulk. The results of stress and temperature were extracted after 2 s. To enable direct comparison among different structures, the computed stress values were normalized by the maximum stress of the two models.

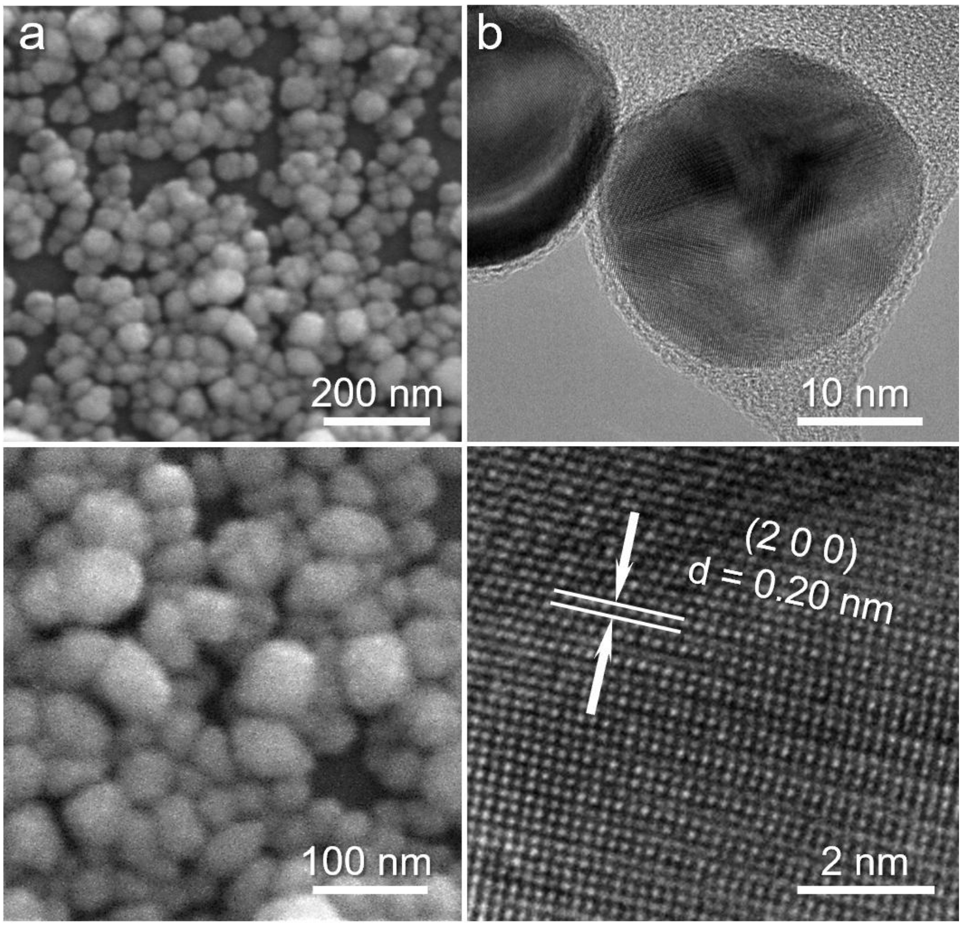

Figure S1. (a, b) SEM (a), TEM and HRTEM (b) images of AgNPs.

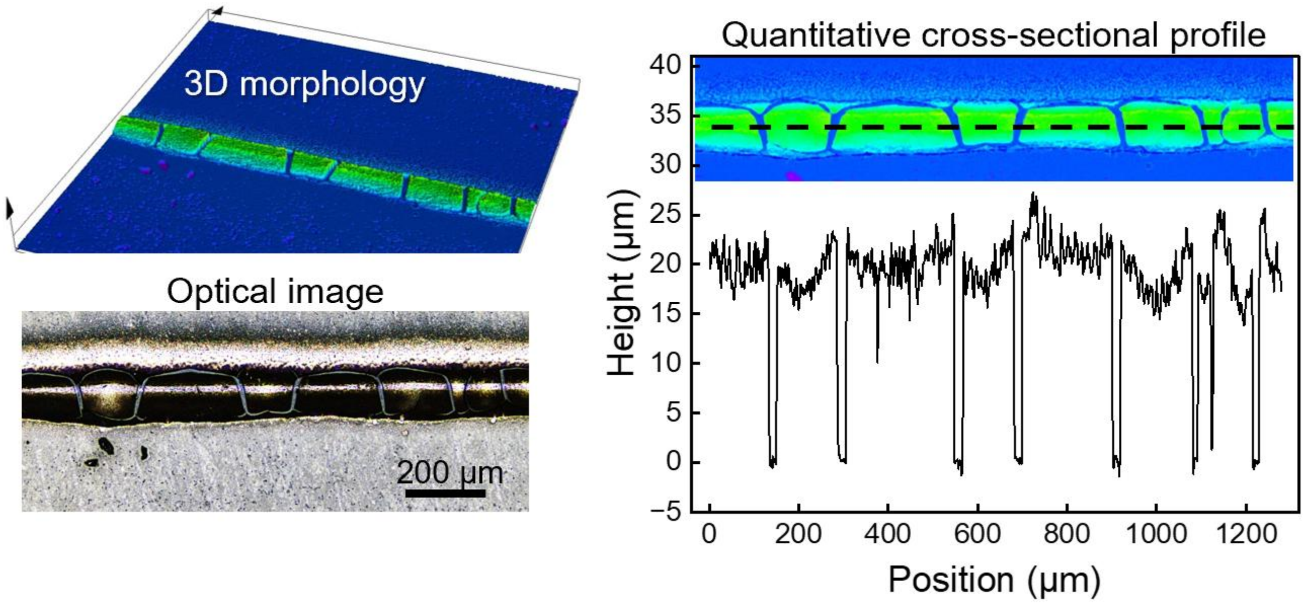

Figure S2. 3D morphology, optical image, and cross-sectional profile of the thick deposit.

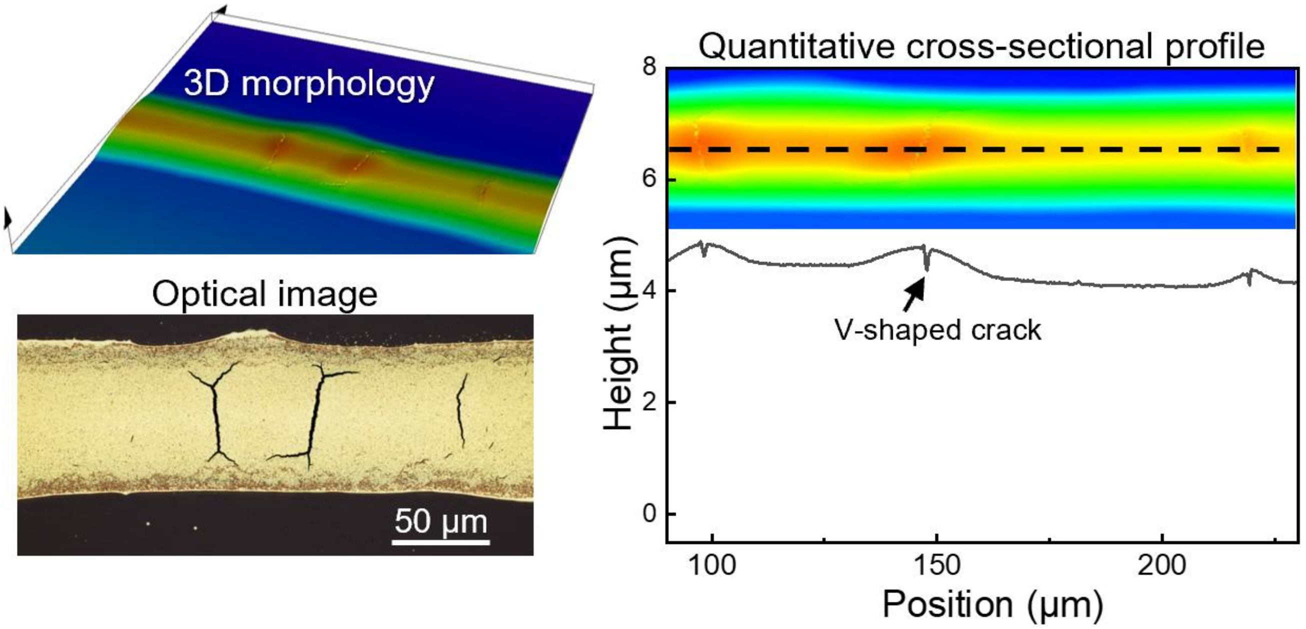

Figure S3. 3D morphology, optical image, and cross-sectional profile of the thin deposit.

The suction stress characteristic theory describes the relationship between the suction stress σ_s (kPa) between soil particles and the moisture content. The derived expression for σ_s via thermodynamics is as follows:

$$\sigma_s = \psi S_e \quad \text{(Equation S1)}$$

To further distinguish between suction stresses generated by capillary action and adsorption, the unified effective stress equation was proposed, differentiating suction stress into capillary suction stress σ_s^{cap} (kPa) and adsorption suction stress σ_s^{ads} (kPa).

$$\sigma_s = \sigma_s^{\text{ads}} + \sigma_s^{\text{cap}} \quad \text{(Equation S2)}$$

For the adsorption suction stress σ_s^{ads}:

$$\sigma_s^{\text{ads}} = f_{\text{ads}}(w)\sigma_s^{\text{dry}} \quad \text{(Equation S3)}$$

where σ_s^{dry} (kPa) is the macroscopic van der Waals attraction between soil particles, and $f_{\mathrm{ads}}(w)$ is a dimensionless scaling function expressed as:

$$f_{ads}(w) = \frac{1}{2}\left[1 - erf\left(\beta \frac{w - w_{tran}^{SS}}{w_{tran}^{SS}}\right)\right] \quad \text{(Equation S4)}$$

where *erf* is the error function, β is a dimensionless parameter reflecting the intensity of adsorption suction stress, w is the gravimetric water content (%), and w_{tran}^{SS} is the transition gravimetric water content (%). For the capillary suction stress σ_s^{cap} :

$$\sigma_s^{cap} = \frac{f_{cap}(w)}{\alpha^{SS}} \frac{w}{w_s} \left[\left(\frac{w}{w_s}\right)^{\frac{n^{SS}}{1-n^{SS}}} - 1\right]^{\frac{1}{n^{SS}}} \quad \text{(Equation S5)}$$

where α^{SS} is the reciprocal of the average capillary suction stress (kPa^{-1}), w_s is the saturated gravimetric water content (%), n^{SS} is a pore size distribution parameter related to capillary suction stress, and $f_{\mathrm{cap}}(w)$ is a dimensionless scaling function expressed as:

$$f_{cap}(w) = \frac{1}{2}\left[1 + erf\left(4 \frac{w - w_{tran}^{SS}}{w_{tran}^{SS}}\right)\right] \quad \text{(Equation S6)}$$

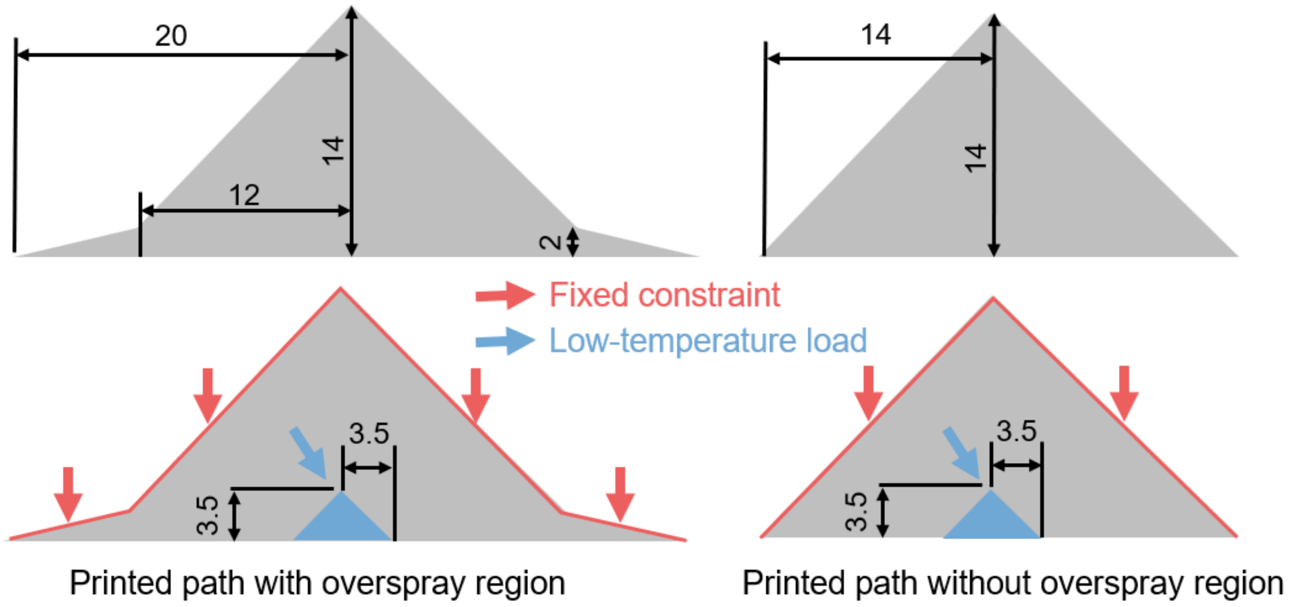

Figure S4. Model dimensions, loads, and constraints for FEA analysis.

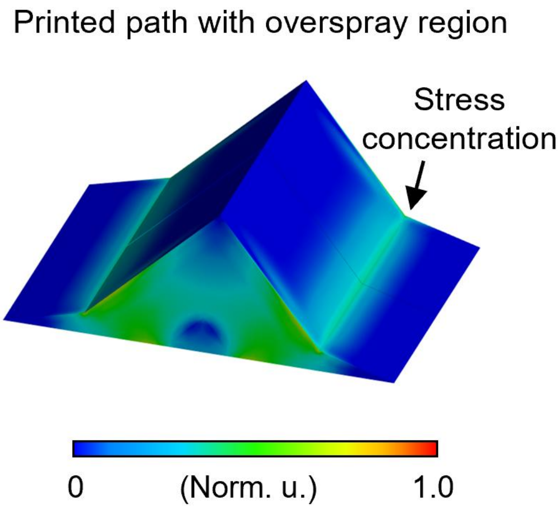

Figure S5. Stress distribution of printed path with overspray region.

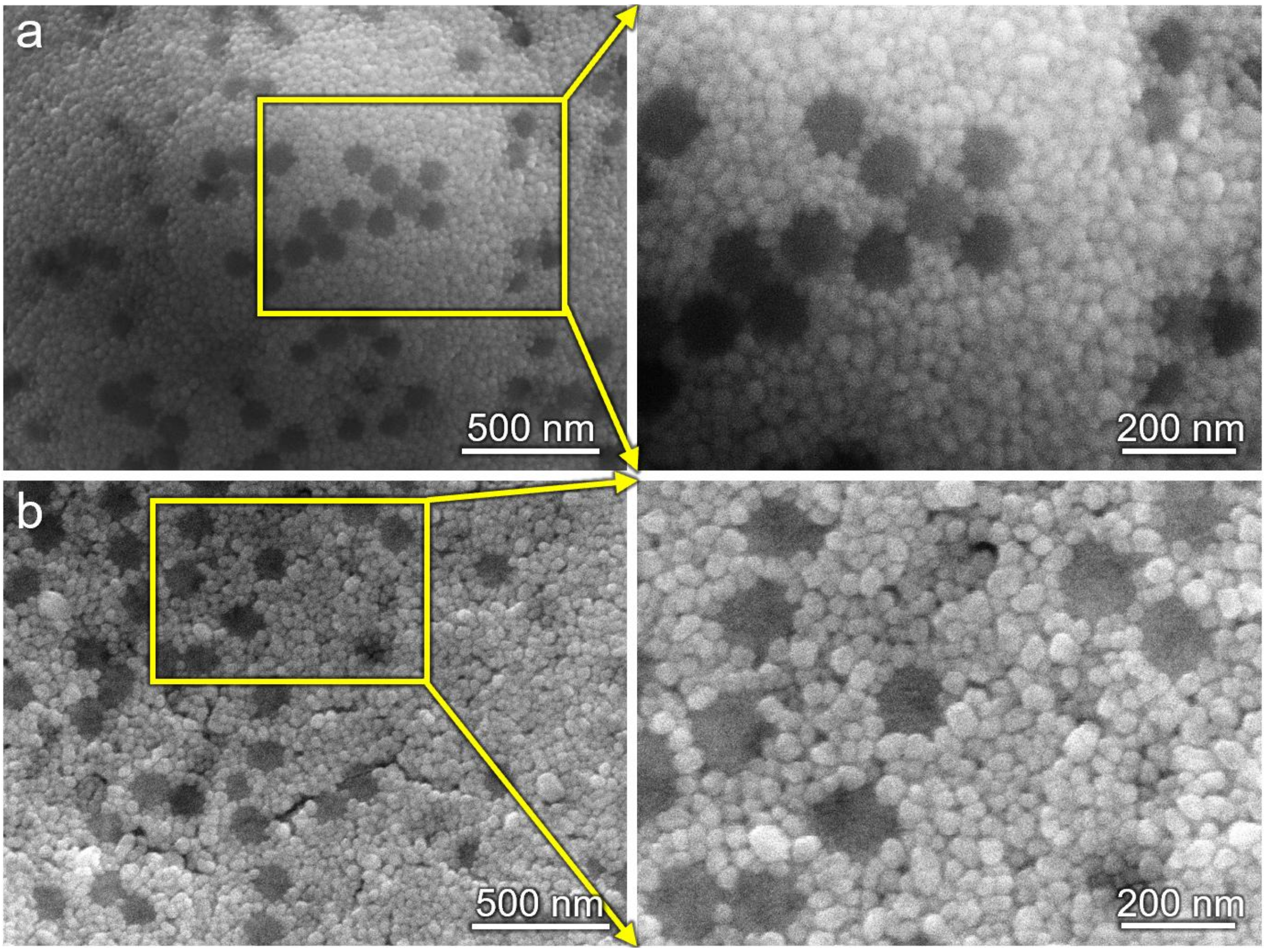

Figure S6. (a, b) SEM images of the printed pattern before sintering.

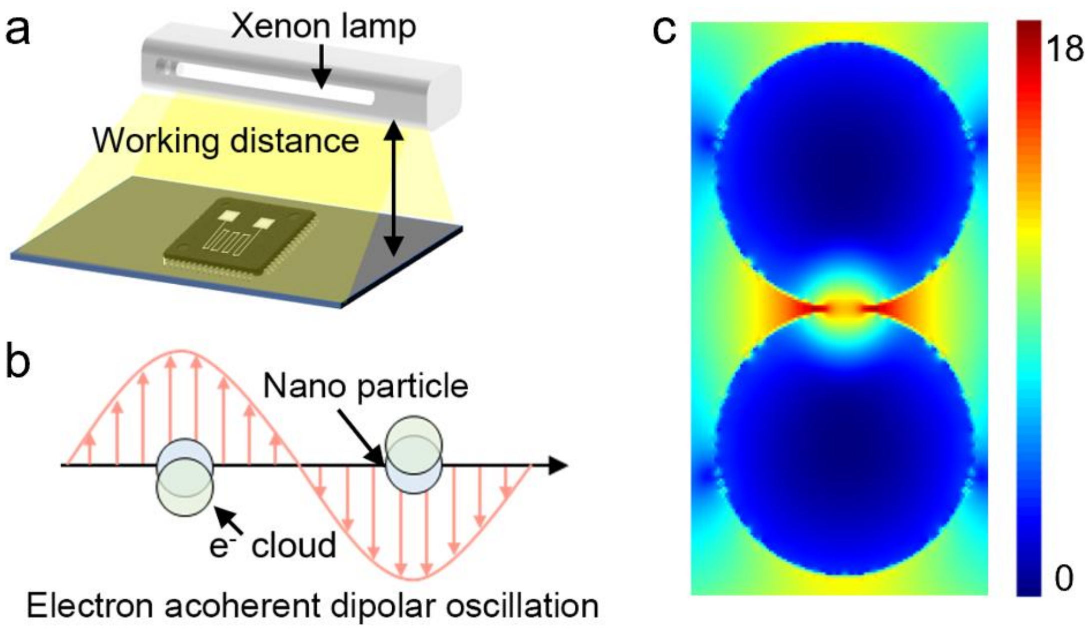

Figure S7. (a-c) Schematic diagrams of PLS (a), electron coherent dipole oscillation (b), and electric field distribution of AgNPs under pulsed light via FDTD simulation (c).

Although thermal sintering is the simplest and most cost-effective sintering method, it is not suitable for temperature-sensitive substrates. The mechanism of PLS is illustrated in Figure S8a-c. The broad-spectrum, high-intensity pulses instantaneously excite the localized surface plasmon resonance within the AgNPs, inducing coherent dipolar oscillations of electrons. This leads to a rapid increase in electron temperature, which is subsequently transferred to the lattice through electron-phonon scattering, efficiently converting optical energy into Joule heat. This process facilitates millisecond-scale sintering between AgNPs without damaging the substrate. The enhancement of the electromagnetic field resulting from the localized surface plasmon effect is the fundamental cause of electron oscillations. Since the nature of pulsed light sintering is still based on thermal effects, the microscopic morphology of the printed patterns varies with sintering voltage in a manner analogous to the change in morphology with sintering temperature and the sintering mechanism in conventional isothermal thermal sintering.

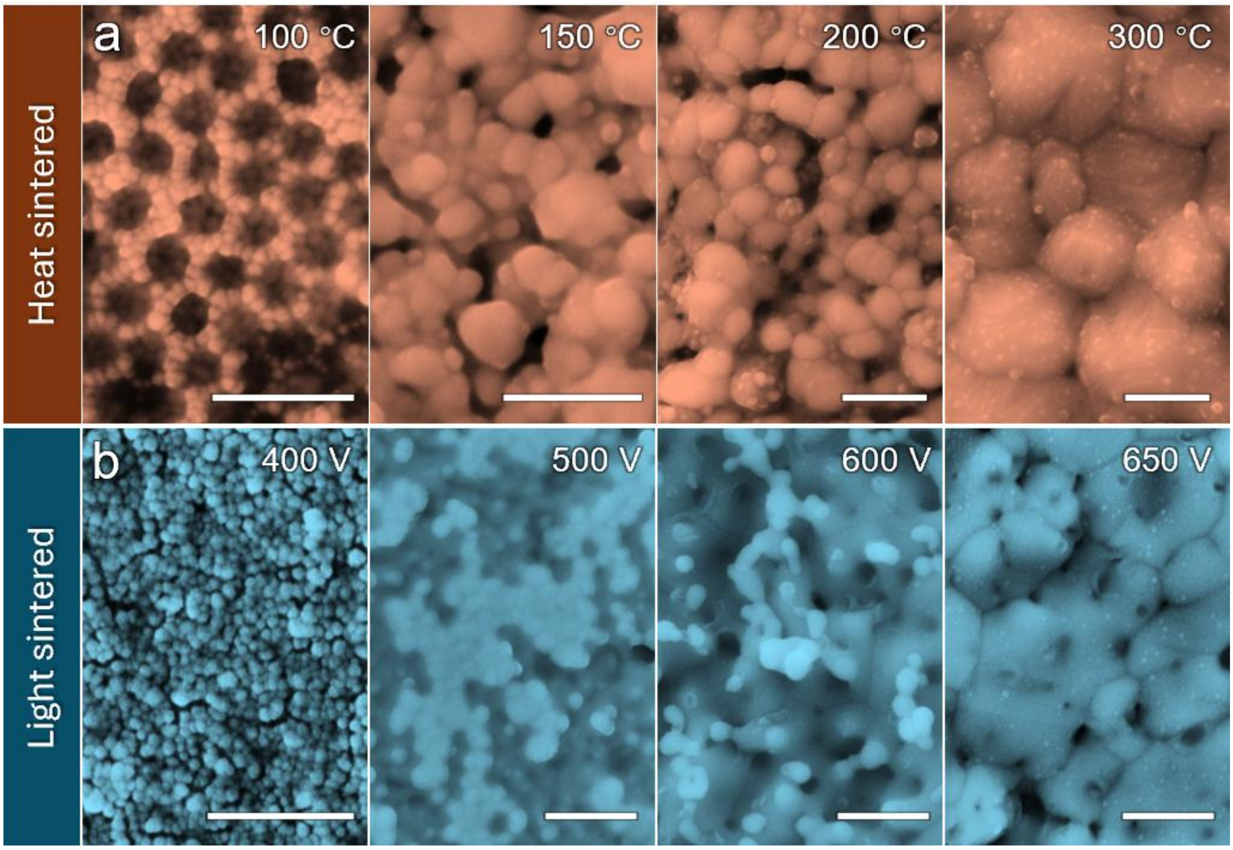

Figure S8. (a, b) SEM images of printed patterns after thermal sintering for 90 min (a) and PLS for 3 ms (b). Scale bars: 500 nm.

Table S1. The resistivity of the printed conductive patterns after thermal sintering

**Thermal sintering**		**Pulsed light sintering**	
Sintering temperature (°C)	Resistivity (μΩ·cm)	Sintering voltage (V)	Resistivity (μΩ·cm)
100	3.1×10^{3}	400	4.8×10^{6}
150	56.6	500	40.5
200	16.2	600	13.4
300	6.0	650	6.8

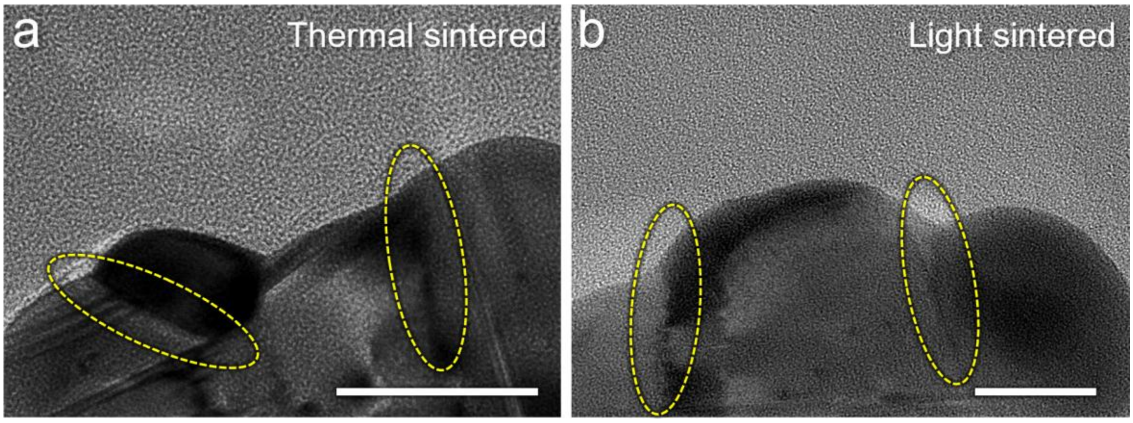

Figure S9. (a, b) TEM images of AgNPs after thermal sintering (a) and PLS (b), with the yellow circle highlighting the sintered interface. Scale bars: 50 nm.

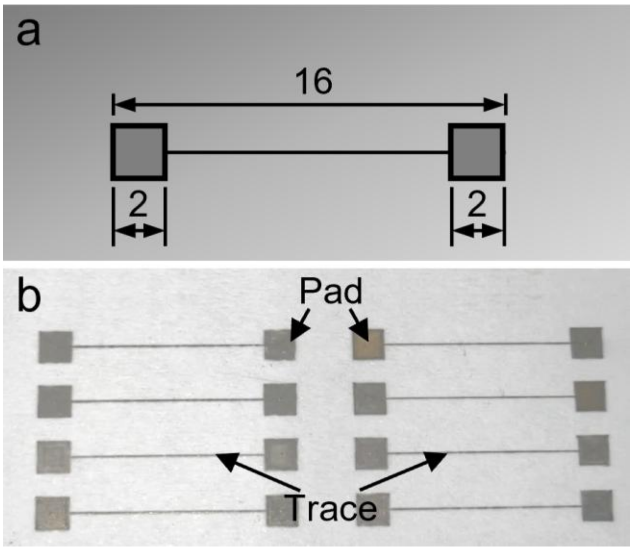

Figure S10. (a, b) The dimensions (a) and optical photographs (b) of the resistivity test samples.

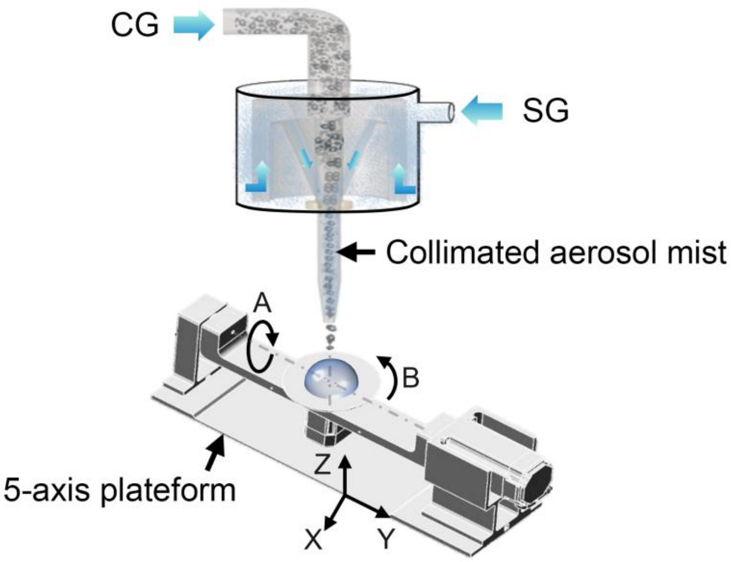

Figure S11. Schematic illustration of AJP on a 3D surface.

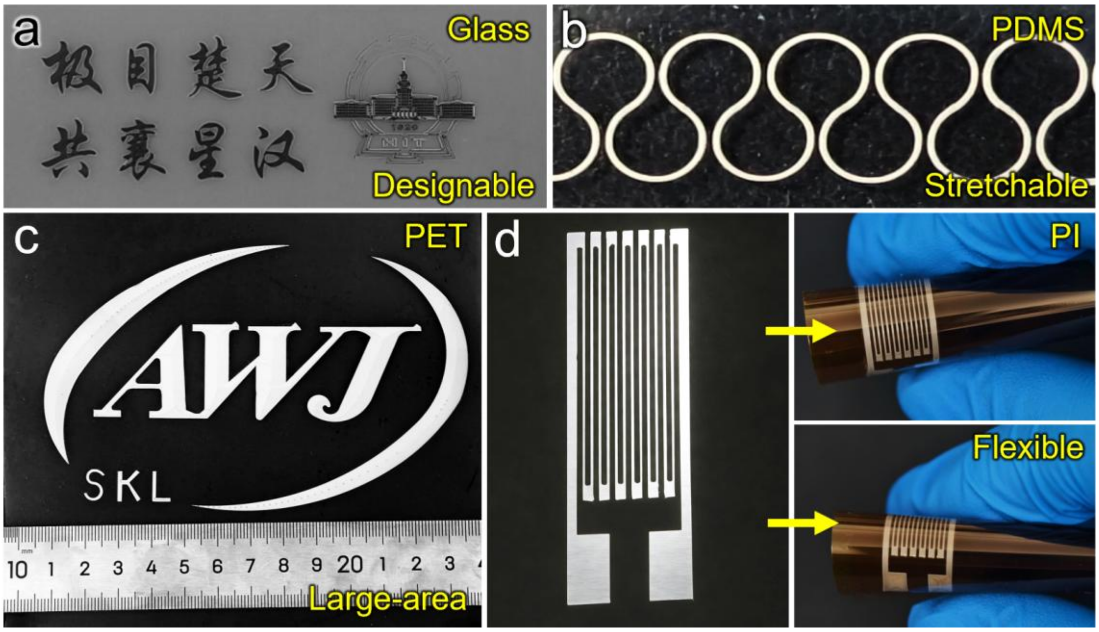

Figure S12. (a-d) Planar patterns printed via AJP on various substrates, exhibiting designable (a), stretchable (b), large-area (c), and (d) flexible characteristics.

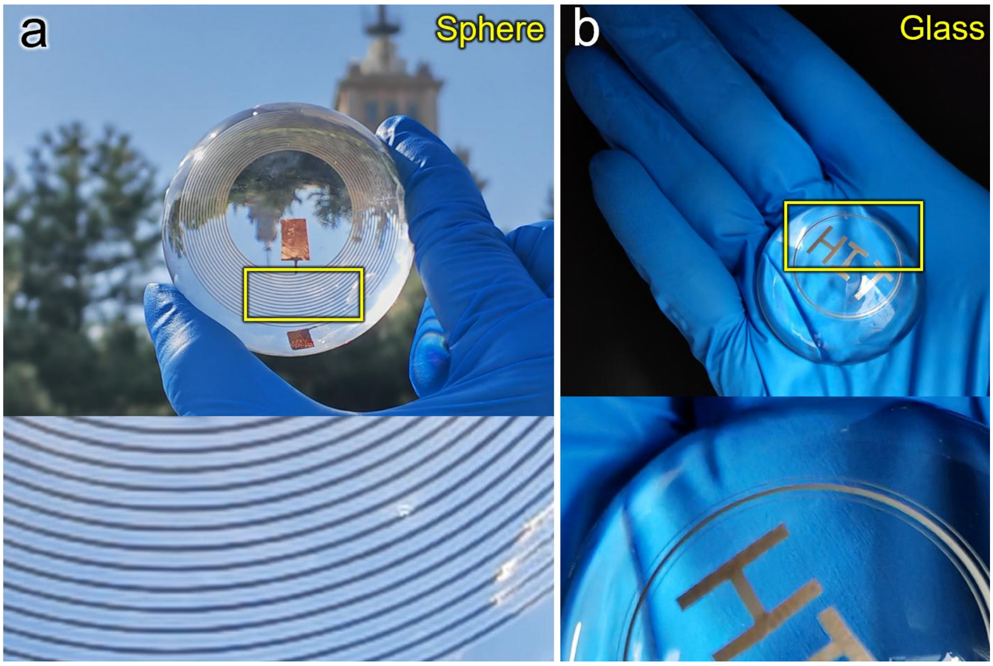

Figure S13. (a, b) Conformal patterns printed on spherical surfaces.

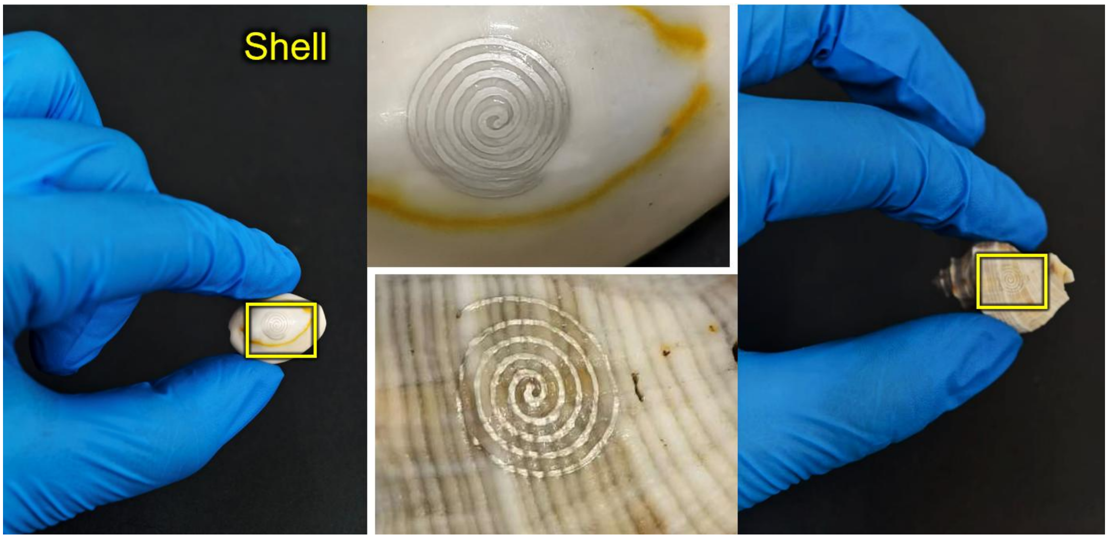

Figure S14. Conformal patterns printed on uneven surfaces.
